# Disruptive behavior scale for adolescents (DISBA): development and psychometric properties

**DOI:** 10.1186/s13034-018-0221-8

**Published:** 2018-03-12

**Authors:** Mahmood Karimy, Ahmad Fakhri, Esmaeel Vali, Farzaneh Vali, Feliciano H. Veiga, L. A. R. Stein, Marzieh Araban

**Affiliations:** 1 Social Determinants of Health Research Center, Saveh University of Medical Sciences, Saveh, Iran; 2 Department of Psychiatry, Ahvaz Jundishapur University of Medical Sciences, Ahvaz, Iran; 3 Institute of Education, University of Lisbon, Lisbon, Portugal; 4 Psychology Dept., University of RI, Kingston, RI USA; 5 Behavioral & Social Sciences Dept., Brown University School of Public Health, Providence, RI USA; 6 RI Training School, Cranston, RI USA; 7 Social Determinants of Health Research Center, Department of Health Education and Promotion, Public Health School, Ahvaz Jundishapur University of Medical Sciences Campus, Golestan BLVD, Ahvaz, 61375-15751 Iran; 8 Department of Health Education and Promotion, Public Health School, Ahvaz Jundishapur University of Medical Sciences Campus, Golestan BLVD, Ahvaz, 61375-15751 Iran

**Keywords:** Adolescence, Disruptive behavior, Validity, Reliability, Psycho-educational development scale

## Abstract

**Background:**

Growing evidence indicates that if disruptive behavior is left unidentified and untreated, a significant proportion of these problems will persist and may develop into problems linked with delinquency, substance abuse, and violence. Research is needed to develop valid and reliable measures of disruptive behavior to assist recognition and impact of treatments on disruptive behavior. The aim of this study was to develop and evaluate the psychometric properties of a scale for disruptive behavior in adolescents.

**Methods:**

Six hundred high school students (50% girls), ages ranged 15–18 years old, selected through multi stage random sampling. Psychometrics of the disruptive behavior scale for adolescents (DISBA) (Persian version) was assessed through content validity, explanatory factor analysis (EFA) using Varimax rotation and confirmatory factor analysis (CFA). The reliability of this scale was assessed via internal consistency and test–retest reliability.

**Results:**

EFA revealed four factors accounting for 59% of observed variance. The final 29-item scale contained four factors: (1) aggressive school behavior, (2) classroom defiant behavior, (3) unimportance of school, and (4) defiance to school authorities. Furthermore, CFA produced a sufficient Goodness of Fit Index > 0.90. Test–retest and internal consistency reliabilities were acceptable at 0.85 and 0.89, respectively.

**Conclusions:**

The findings from this study suggest that the Iranian version of DISBA questionnaire has content validity. Further studies are needed to evaluate stronger psychometric properties for DISBA.

## Background

Adolescence is considered one of the major periods in structuring and establishing the personality [1]. Further, it is a crucial time in which mental and behavioral disorders may manifest [2, 3]. Early diagnosis and timely intervention of adolescents with mental and behavioral disorders is very important [4], and since 1950 many studies have been carried out on the prevalence of behavior disorders and problems among student adolescents [5, 6]. It is likely that the behavior problems which arise in this period appear later on in life as stable characteristics; therefore, detection of such behavior among students, and dealing with them correctly is essential [7].

In particular, disruptive behavior disorders (DBDs), including conduct disorder (CD), oppositional defiant disorder (ODD) and attention deficit hyperactivity disorder (ADHD), may manifest in children and adolescents and can be associated with a host of school difficulties and problems in later life. Common symptoms occurring in individuals with CD and ODD include: defiance of authority figures, angry outbursts, and other antisocial behaviors such as lying and stealing. It is felt that the difference between oppositional defiant disorder and conduct disorder is in the severity of symptoms and that they may lie on a continuum often with a developmental progression from ODD to CD with increasing age [8]. Furthermore, ODD often includes problems of emotional dysregulation (i.e., angry and irritable mood) not included in definitions of CD (American Psychiatric Asso., 2013) [9].

Today, there is little doubt regarding the emergence of disruptive behavior in adolescence. According to surveys, two to six percent of adolescents from typical demographics of society have some level of disruptive behavior [10]. This behavior has caused concern for families, schools, and public health, constituting the most common reason for adolescents to visit psychiatric clinics [7]. Students with disruptive behavior are faced with educational problems such as academic failure, expulsion, dropping out, and low grades, as well as high-risk behavior such as drug and alcohol abuse, and high-risk sexual behavior [11].

Students with disruptive behavior interrupt the learning process for other students, and the teacher’s ability to teach effectively; they also divert school resources and energy away from the main academic goals [12]. Adolescents with disruptive behavior have problems with their ability to understand and manage emotions [13] and higher risk of committing anti-social and criminal behavior [14]. Disruptive behavior is behavior which truly disrupts the learning and teaching processes in classroom or any other educational environment [15–17].

The cause of DBDs is not known. DBDs are more common among children aged 12 years and older; and child abuse or neglect and a traumatic life experience have been stated as risks for DBDs [18]. Additionally, It has been documented some socio-psychological and cultural factors may contribute to disruptive behavior [19]. For example, parent–child and school-child relationships may enhance the risk of developing DBD [20]; it has also been shown that life satisfaction and hope are negatively related to adolescent problem behaviour [21]. Disruptive behavior disorders are associated with psychological problems including anxiety, depression [22] and development of antisocial personality disorder later in life [19]. Psychosocial interventions that include parents, children, families and teachers, as well as behavioral support, can improve this disorder among adolescent [23].

DBD’s are related to poor outcomes for youth including involvement in crime and numerous educational problems [16, 17]; therefore, timely screening, detection and management of DBDs are of critical importance [24]. A number of rating scales exist to assist in detecting DBDs including the Conner’s Parent Rating Scale (CPRS), the disruptive behavior rating scale (DBRS), and the disruptive behavior scale-professed by students (DBS-PS). The CPRS focuses mainly on ADHD [8]; the DBRS consists of 45 questions, and has been validated among young children [25]; and the DBS-PS has only been validated among Portuguese students [15]. It is advantageous to develop screening that encompasses DBDs more generally, is brief, and is of relevance to older children. Furthermore, expanding validation of such screening tools to cultures other than Western cultures is important.

Because DBDs are association with important and potentially life-long impairment, and because DBDs are associated with significant societal costs, the current study aimed to design a suitable scale for screening DBDs. We therefore, designed a 29-item disruptive behavior screening scale among Iranian high school students and analyzed its psychometric properties.

## Methods

The research population consisted of all the high school students aged fifteen to eighteen of Saveh city in the academic year 2015–2016. The sample size was determined four hundred people, considering the number of items in the scale (initially, 39 items) and following Munro [26] who recommended ten people for each item. To increase the accuracy of the study, six hundred students (300 girls and 300 boys) were selected for the study.

### Sampling

A multi stage sampling was applied. Firstly, Saveh-a city located in the center of Iran, was divided into two parts: north and south. Among all high schools located in each region, four high schools (2 girls high schools and 2 boys high schools) were randomly selected from each district, which constituted a total of eight high schools. Then, among all students attending a high school, a random sample was selected using random numbers. It is worth mentioning that the required sample ratio for participation in the study was determined for each high school according to the number of students in each high school. In the last stage, the ratio of samples participating in the study from each class was determined for each of first and fourth grades according to the number of students in each grade.

The students were 15 to 18 years old and were the tenth, eleventh and twelfth grade students.

The students answered the anonymous scale without the presence of teachers, and without any compulsion, in a self-administered manner.

### The scale

A student’s disruptive behavior scale was developed for this study. The disruptive behavior scale for adolescents (DISBA) was designed in reference to literature review and semi-structured interviews with students and high school authorities. Disruptive behavior in this study was considered as any type of behavior, which truly disrupts the learning and teaching processes in classroom or educational environment.

To develop the item-pool, we considered previous scales on disruptive behavior and conducted semi-structured interviews. The initial item pool consisted of 39 items, including the 16-item DBS-PS [15] along with 23 items derived from literature review [8, 24, 25] and interviews.

To develop the 23 items, focus groups were conducted with thirty students who were similar to the target population in terms of demographic properties, as well as with ten teachers and school staff Focus group data were analyzed for thematic content and then a panel of experts developed 23 items based on focus group themes and the literature. Finally, the research team then decided to utilize a 4-point Likert scale response option consisting of never (0), rarely (1), usually (2) and always (3) for each item.

### Statistical analysis

#### Face validity

Both qualitative and quantitative methods were used to determine face validity. For quantitative face validity, 20 students were asked about the importance of each item in helping to identify disruptive classroom behavior. For the qualitative approach, students were asked to assess each item for ambiguity and difficulty. Overall, no problems in reading or understanding the items were expressed by the students. The quantitative face validity was evaluated through item impact score. Participants were asked to rate the importance of each item on a five-point Likert type scale form strongly important to not at all important. The scores ranged from 1 to 5 for each item. The item impact score for each item was calculated by multiplying the mean score of importance of an item with its frequency by relative frequency (percentage). The item impact scores of greater than 1.5 were considered suitable.

#### Content validity

A panel of experts (15 specialists in health education, psychiatry, health psychology, and educational psychology) rated items according to relevance. Each item was rated according to the following: (1) irrelevant, (2) important, but not essential, (3) essential. For each item a Content Validity Ratio (CVR) was computed as (n_e_ − N/2)/(N/2), where n_e_ is the number of experts rating the item as essential and N is the number of experts. The overall CVR index of the scale is computed as a mean of the items’ CVR values. The Content Validity Index (CVI) was also calculated Experts rated items on a four-point rating scale: (1) not relevant, (2) somewhat relevant, (3) quite relevant, and (4) very relevant. CVI is the percentage of experts rating an item as quite or very relevant. The recommended value for CVR is 0.59, for CVR scale index it is determined using Lawshe’s table, and for CVI the minimum recommended value is 0.79 [27, 28].

#### Construct validity

Exploratory factor analysis (EFA) was carried out to identify the underlying relationships between items. To determine the adequacy of the sample size, a Kaiser–Meyer–Olkin test was applied. A threshold of > 0.5 for corrected item–total–correlation was chosen as sufficient. SPSS 15 (SPSS, Inc., Chicago, IL, USA) was utilized for analyses, and items with factor loadings over 0.50 were retained. Confirmatory Factor Analysis (CFA) was carried out to test whether the data fit the hypothesized measurement model. The following cut-offs were considered appropriate [29]: 0.90 for the Comparative Fit Index (CFI), Goodness of Fit Index (GFI) and Normed Fit Index (NFI), 0.08 for the root mean square error approximation (RMSEA). Lisrel 8.8 (Scientific Software International, Inc., 2007) was used in this study for confirmatory factor analysis.

#### Reliability

Two methods were used to assess reliability: internal consistency and stability as described below:

1. Internal consistency: this was assessed using Cronbach’s alpha coefficient. The value of 0.7 or above was considered satisfactory [30].
2. Test–retest analysis. N = 25 students from the study sample completed the scale twice with an interval of 2 weeks. The intraclass correlation coefficient (ICC) was calculated and a value of 0.4 or above was considered acceptable [30, 31].

### Ethics statement

The study was approved by the Ethics Committee of Saveh University of Medical Sciences. All the participants had signed the informed written consent form, where the confidentiality of the information received and the anonymity of responses to the scales was stressed.

## Results

The average age and its standard deviation was 16.83 ± 0.86 for the male students and 16.62 ± 0.85 for the female students. The grade point average (GPA) of students was 15.8 ± 2.3 in the year before, on a scale from 12 to 20, where 20 indicates better performance. One hundred fifty-nine students (26.5%) had a history of smoking cigarettes or hookah within the 7 days before completing the study. One hundred fifty-three students (25.5%) were not happy with their lives.

Seven questions were omitted through examination of CVR, while three questions were omitted through examination of CVI. Twenty-nine out of thirty-nine questions, which had proper content validity, entered the stage of construct validity assessment using exploratory factor analysis. The Kaiser–Meyer–Olkin test for sampling adequacy and Bartlett’s sphericity test both indicated the data were suitable for EFA.

In the next stage, the Exploratory Factor Analysis found four factors with Eigen values greater than one: (1) aggressive school behavior, (twelve questions), (2) classroom defiant behavior (six questions), (3) unimportance of school, (six questions), and (4) defiance to school authorities (five questions). The factor loading matrix in Table 1 shows that all the extracted factor loadings are greater than 0.50, and these factors explain a total of fifty-nine percent of the cumulative variance.

**Table 1:** The result obtained from exploratory factor analysis with varimax rotation among adolescents aged 15–17 (n = 600)

Item	Factor 1	Factor 2	Factor 3	Factor 4
I hit the school trees and break their branches	0.722^a^			
I stick gum on the seats	0.668			
I love to carve on the school benches	0.611			
I tuck the back of my shoes like villains when I walk	0.610			
I sometimes come to school after taking drugs	0.606			
I bring explosives to school	0.600			
I deliberately break or damage school equipment	0.589			
I get expelled from class due to inappropriate and disruptive behavior	0.562			
I like to drag my feet when I walk	0.558			
I text messages in class while the teacher is teaching	0.549			
I kick the classroom door open	0.536			
I clash with teachers	0.503			
I make noise and disrupt the class		0.622		
I eat refreshments in class without permission		0.621		
I like to disrupt the class and the school		0.611		
I speak without permission and disrupt the class		0.594		
I argue with my classmates		0.570		
I sing out loud at school		0.543		
I don’t turn up on time for school			0.711	
I turn up late for class			0.655	
I forget to bring the things I need to school			0.560	
I don’t pay attention to the lessons in the classroom			0.532	
I skipping classes			0.530	
I can’t relate well with my friends			0.511	
I don’t care about school’s teachers and authorities				0.650
I argue with teachers				0.640
I leave my seat without teacher’s permission				0.607
I argue with the school’s authorities				0.543
I don’t stand up when the teacher enters the class				0.541
Total variance explained	59%			

^a^Factor loadings less than 0.3 were omitted

Confirmatory factor analysis was carried out to assess the results from the Exploratory Factor Analysis. The results showed that the structural model provided a good fit to the data. The Chi square value was significant χ^2^ = 17.16, df = 7.4, p = 0.02). The Goodness-of-Fit Index was 0.91, the adjusted goodness-of-fit index was 0.90, the Normed Fit Index was 0.92, the Comparative Fit Index was 0.96, and the root mean square error of approximation as 0.05. These figures indicate that the four-factor model of disruptive behavior has satisfactory goodness-of-fit (Table 2).

**Table 2:** The results obtained from confirmatory factor analysis

RMSEA	GF	NF	CFI	d	χ^2^
0.05	0.91	0.92	0.9	7.4	17.16

*RMSEA* root mean square error of approximation; *GFI* goodness-of-fit index; *NFI* Normed Fit Index; *CFI* Comparative Fit Index

The reliability of the scale was assessed in terms of internal consistency and temporal stability. The Cronbach’s alpha coefficient ranged from 0.77 to 0.91, ICC’s ranged from 0.71 to 0.88 indicating satisfactory stability (Table 3).

**Table 3:** Cronbach’s α coefficient and ICC for the disruptive behavior scale and its subscales

Domain	Number of items	Cronbach’s α coefficient	ICC
Aggressive school behavior	12	0.82	0.79
Classroom defiant behavior	6	0.91	0.87
Unimportance of school	6	0.77	0.71
Defiance to school authorities	5	0.86	0.88
Total scale	29	0.89	0.85

*ICC* intraclass correlation coefficient

## Discussion

It is critical to detect students who may have disruptive behavior disorder, given that such behavior may lead to high-risk behavior such as delinquency, violence, drug abuse and anti-social personality if left untreated [28, 30]. This study presents a brief, valid and reliable scale with sub-parts that may aid in screening for DBDs in youth. Furthermore, many such scales are primarily created and validated in Westernized cultures, and it is important to expand validation and use in other countries.

To study construct validity, factor analysis was used and showed a 4-factor construct that explained 59% of variance, which is consistent with other similar studies [32]. Results of confirmatory factor analysis show that the data with the four presented constructs have sufficient goodness-of-fit.

The four-factor structure is not consistent with results obtained for the DBS-PS which yielded a three-factor structure consisting of distraction-transgression, schoolmate aggression and aggression to school authorities [15]. The four-factor structure found in this study included (1) aggressive school behavior, (2) classroom defiant behavior, (3) unimportance of School, and (4) defiance to school authorities. One possible explanation for such a difference at factor-level may be due to the fact that these scales have different number of items and have been validated among different populations with different age ranges and cultural backgrounds.

Based on DSM-5 disruptive behavior and ADHD are two distinct disorders although they may present similarly and may be co-exist. Behavior of children with ADHD may be disruptive, but this behavior by itself does not violate social norms or others’ rights and so does not usually meet criteria for CD [9]. As such, there are similarities between DISBA and scales that screen for ADHD symptoms including: losing things, making mistakes, arguing, damaging things or equipment, failing to do tasks, having problem with relationship and skipping schools. While screening can alert professionals to a potential behavioral problem, further assessment and diagnosis will help in determining how to target and tailor interventions for specific disorders.

The results of the study show that the students’ disruptive behavior scale has good internal consistency ranging from 0.77 to 0.91. This is consistent with a similar study on Portuguese students that also showed the reliability ranged from 0.67 to 0.88 [15]. Test–retest results indicate a high degree of reliability in the DISBA, which is again consistent with the aforementioned study on Portuguese students that found test–retest reliability to be 0.85 [15].

## Limitation

Future studies may wish to examine the correlations between scales and other phenomena associated with DBD, such as observations of stealing, fighting, etc. In addition, future studies may wish to examine how well scales distinguish between youth with and without a diagnosis of DBD (ODD, CD, or ADHD), and sensitivity to detect change in behavior over time (e.g., following intervention).

## Conclusion

According to the results of this study, this brief 29-item scale evidences good validity and reliability. School authorities and teachers might use DISBA to screen students in order to identify problematic students in need of further evaluation for diagnosis and intervention. Although based in part on the DBS-PS, the DISBA evidences good psychometrics in a non-Westernized culture, allows for screening based on four relevant scales for the school setting as compared to only three, and can be used with youth ages 15–18 years old.

## Abbreviations

CVR: content validity ratio
CVI: Content Validity Index
CFA: confirmatory factor analysis
RMSEA: root mean square error of approximation
NFI: Normed Fit Index
GFI: Goodness of Fit Index
CFI: Comparative Fit Index
